# Gene Expression of VEGF-A and VEGF-C in Peripheral Blood Mononuclear Cells of Iranian Patients with Acute Myeloid Leukemia

**DOI:** 10.4274/Tjh.2011.0023

**Published:** 2013-06-05

**Authors:** Mohammad Reza Aliparasti, Shohreh Almasi, Zohreh Sanaat, Aliakbar Movasaghpoor, Reza Khalili-Dizaji, Homayoun Sadeghi-Bazargani

**Affiliations:** 1 Hematology and Oncology Research Center, Tabriz University of Medical Sciences, Tabriz, Iran; 2 Immunology Research Center, Tabriz University of Medical Sciences, Tabriz, Iran; 3 Department of Immunology, Faculty of Medicine, Tabriz University of Medical Sciences, Tabriz, Iran; 4 Neuroscience Research Center, Department of Statistics & Epidemiology, School of Health & Nutrition, Tabriz University of Medical Sciences, Tabriz, Iran

**Keywords:** Acute myeloid leukemia, VEGF-A, VEGF-C, Gene expression, Angiogenesis

## Abstract

**Objective:** The crucial role of angiogenesis in the pathophysiology of acute myeloid leukemia (AML) has been proposed. One of the key regulators of angiogenesis is the vascular endothelial growth factor (VEGF). Among the VEGF family, it has been observed that VEGF-A and VEGF-C are expressed by AML cells and mediate leukemic cell proliferation, survival, and resistance to chemotherapy. Emerging evidence, however, suggests that elevated levels of VEGF or a proangiogenic phenotype may impede, rather than promote, early tumor development and progression. As the significance of VEGF-A and VEGF-C levels in the pathogenesis of AML has not been clarified well, the aim of this study is to evaluate gene expression of these angiogenesis promoters and its possible prognostic value in peripheral blood mononuclear cells of Iranian patients with AML.

**Materials and Methods:** We investigated the mRNA expression of VEGF-A and VEGF-C in peripheral blood mononuclear cells of 27 patients with newly diagnosed AML and 28 healthy controls by quantitative real-time PCR.

**Results:** Expression of VEGF-C mRNA was significantly lower in AML patients than in healthy controls (p<0.001). However, there was no significant decrement in expression of VEGF-A mRNA of AML patients compared to the control group (p=0.861). VEGF-A and VEGF-C expression were not able to predict clinical outcome.

**Conclusion:** Our data showed that AML is associated with a decreased expression of VEGF-C mRNA. However, expression levels did not influence the clinical outcome in our study. It seems that angiogenesis is affected by different cytokines other than VEGF-C or VEGF-A, and VEGF is also affected by different cytokines. Taken together, these findings help to provide new insights into the investigation of other angiogenic factors and cytokines that may play roles in the pathogenesis of AML.

**Conflict of interest:**None declared.

## Abstract (tr)

**Amaç:** Akut miyeloid lösemi (AML) patofizyolojisinde anjiyogenezinönemli rol oynadığı ileri sürülmektedir. Anjiyogenezdeki anahtar düzenleyicilerden biri damar endotel büyüme faktörü- vascular endothelial growth factor (VEGF)- dür. VEGF ailesi içinde, VEGF-A ve VEGF-C’nin AML hücreleri tarafından eksprese edildiği ve lösemik hücrenin proliferasyonu, yaşamı ve kemoterapiye direncine aracı olduğu gözlenmiştir. Buna rağmen mevcut bilgiler, artmış VEGF düzeylerinin veya proanjiyogenik bir fenotipin erken tümör gelişimi ve progresyonunu tetiklemekten ziyade engelleyebildiğini göstermiştir. VEGF-A ve VEGF-C düzeylerinin AML patogenezindeki yeri tam olarak açıklanamadığından, bu çalışmanın amacı İranlı AML hastalarının periferik kan mononükleer hücrelerinde sözügeçen anjiyogenez düzenleyicilerinin gen ekspresyonlarını ve prognostik değerini incelemektir.

**Gereç ve Yöntemler:** Yirmi yedi yeni tanı AML hastası ile 28 sağlıklı köntrolün periferik kan mononükleer hücrelerinde kantitatif real-time PCR ile VEGF-A ve VEGF-C’nin mRNA ekspresyonu araştırdık.

**Bulgular:** AML hastalarındaki VEGF-C mRNA ekspresyonu sağlıklı kontrollere göre belirgin olarak düşüktü (p<0.001). Buna karşılık VEGF-A mRNA ekspresyonunda kontrol grubuna göre anlamlı bir azalma yoktu (p=0.861). VEGF-A ve VEGF-C ekspresyonunun klinik sonucu ön görme kapasitesi yoktur.

**Sonuç:** Bulgularımız AML’nin azalmış VEGF-C mRNA ekspresyonu ile ilişkili olduğunu gösterdi. Buna rağmen ekspresyon düzeyleri bizim çalışmamızda klinik sonucu etkilemedi. Hem VEGF başka sitokinlerden hem de anjiyogenez VEGF-C veya VEGF-A’dan başka sitokinlerden etkileniyor gibi görünmektedir. Sonuç olarak, bulgularımız AML patogenezinde rolü olabilecek diğer anjiyojenik faktör ve sitokinlerin araştırılmasına yeni bir bakış açısı sağlayarak yardımcı olacaktır.

## INTRODUCTION

Acute myeloid leukemia (AML) is an aggressive hematologic malignancy characterized by accumulation of immature malignant myeloid cells in the bone marrow and blood due to their clonal proliferation without substantial maturation [1,2]. The pivotal role of angiogenesis has been suggested in the pathophysiology of AML. The crucial role of angiogenesis in the growth, persistence, and metastases of solid tumors has been indicated in many studies [3,4]. Moreover, the importance of angiogenesis in the pathogenesis of hematologic malignancies has been recognized recently. Earliest studies have reported the increased microvessel density in the bone marrow of AML patients compared to normal groups [5,6,7,8]. Angiogenesis is controlled by a balance between proangiogenic and antiangiogenic growth factors and cytokines [9,10]. One of the most key regulators of angiogenesis is the vascular endothelial growth factor (VEGF), which increases permeability and promotes proliferation, migration, and differentiation of endothelial cells. The most responsible factor for angiogenesis is hypoxia, which induces expression of VEGF [10]. The VEGF family includes 5 glycoproteins: VEGF-A, VEGF-B, VEGF-C, VEGF-D, and PGF [11]. Among the VEGF family, it is known that VEGF-A and VEGF-C are expressed by AML cells [12,13].

There are 3 known VEGF receptor tyrosine kinases, VEGFRs 1, 2, and 3, that are exclusively expressed in endothelial cells, hematopoietic stem cells, and tumor cells [14,15]. It has been observed that leukemia cells commonly express one or both of VEGFR-1 and VEGFR-2, and they can produce and secrete VEGF [16,17]. It has been shown that VEGF stimulates a mitogenic response in hematologic malignancies and promotes self-renewal of leukemia progenitors [17,18]. The role of VEGF-A as a proangiogenic factor in AML has been well documented [19]. Furthermore, recent studies have revealed the contribution of VEGF-C in hematological malignancies’ progression [20,21,22]. However, in spite of the evidence of the angiogenic role of VEGF in AML, there are investigations that reported lower VEGF-C [23,24] and VEGF-A [23] expression in the AML patient’s bone marrow than in healthy controls. Therefore, as the significance of VEGF-A and VEGF-C levels in the pathogenesis of AML has not been clarified well, the aim of this study was to evaluate gene expression of VEGF-A and VEGF-C and its possible prognostic value in peripheral blood mononuclear cells (PBMCs) of Iranian patients with AML.

## MATERIALS AND METHODS

**Patients and Controls**

Twenty-seven (14 female and 13 male) patients with newly diagnosed AML who were referred to Shahid Ghazi Tabatabai Hospital in Tabriz from September 2009 to July 2010 were enrolled in this study. The initial diagnosis of AML and its subtypes were determined according to the French-American-British classification [25]. AML smears were routinely investigated at the same hospital, and subtyping was confirmed by flow cytometry. The clinical data of these patients are summarized in Table 1. The control group consisted of 28 healthy volunteers (14 females and 14 males) who were ethnically, age-, and sex-matched to the patients and were recruited from the Tabriz Blood Transfusion Organization. Control subjects were screened by a physician using a questionnaire to ensure the absence of any hematological malignancies as well as a personal or family history of AML. This study was approved by the local ethics committees.

**Blood Sampling and PBMC Isolation**

EDTA-added whole blood was collected from AML patients and controls. Mononuclear cells were isolated from peripheral blood by Ficoll-Hypaque density gradient centrifugation.

**RNA Extraction and First-Strand cDNA Synthesis**

Total RNA was extracted from PBMCs and blasts using Trizol Reagent (Invitrogen, USA) according to the manufacturer’s description and treated with RNase-free DNase to remove any residual genomic DNA. Single-stranded cDNAs were synthesized by incubating total RNA (1 μg) with RevertAid H Minus M-MuL V reverse transcriptase (200 U), oligo-(dT)18 primer (5 μM), random hexamer primer (5 μM), dNTPs (1 mM), and RiboLockRNase-inhibitor (20 U) for 5 min at 37 °C, followed by 5 min at 25 °C followed by 60 min at 42 °C in a final volume of 20 μL. Reaction was terminated by heating at 70 °C for 5 min.

**Real-Time Relative Quantitative RT-PCR**

Quantitative real-time PCR was done using the Corbett Life Science System (Rotor-Gene 6000) with 2 μL of 4-fold diluted cDNA in each PCR reaction in a final volume of 20 μL. Each PCR reaction contained 150 nM of primers and 1X FastStart SYBR Green Master (Roche). Sequences of primers are listed in Table 2. PCR amplifications were performed by the following 3-cycle program: 1) denaturation of cDNA (1 cycle: 95 °C for 10 min); 2) amplification (40 cycles: 95 °C for 15 s, 57 °C for 30 s, 60 °C for 34 s); 3) melting curve analysis (1 cycle: 60 to 95 °C with temperature transition rate of 1 °C/s). β-Actin (ACTB) mRNA expression levels were used to calculate relative expression levels. All data are presented as a ratio of the target gene/ACTB. The relative quantification was performed by 2(−ΔCt): expression of target genes / β-actin = (1+E)-Ct target gene / (1+E)-Ct β-actin.

The specificity of the PCR reactions was verified by generation of a melting curve analysis followed by gel electrophoresis, visualized by ethidium bromide staining.

**Standard Curve**

Efficiency of RT-PCR reactions was determined by a standard curve, which was derived from the 10-fold serial dilution of a positive PCR product by a customary RT-PCR. Logarithms of concentrations were plotted against the target gene cycling threshold (Ct) of serial dilution. VEGF-A, VEGF-C, and ACTB efficiencies were 95%, 91%, and 96% respectively.

**Statistical Analysis**

Expression of VEGF-A and VEGF-C was obtained with the Corbett Rotor-Gene 6000.

Normal distribution of data was evaluated using Stata software with qnorm program version 11. Data were analyzed with SPSS 16. Variables that had normal distribution were reported as means and standard deviations. Medians were reported for the variables whose distribution deviated from the normal distribution. Differences between diagnostic groups were evaluated using the Kruskal–Wallis test and comparisons of gene expression levels between AML patients and the control group were performed with the Mann–Whitney test. Overall survival was measured from the date of first diagnosis to death from any cause. Kaplan–Meier estimation was used to plot survival curves, and log-rank tests were used to test the difference between groups. Univariate and multivariate Cox regression analyses were also used to estimate prognosis. Proportional hazard assumption was checked for the survival models. The correlation between continuous variables was studied using Spearman’s rank correlation (rs). All tests were 2-tailed and a 5% significance level was applied.

## RESULTS

**Patients and Controls**

We studied 27 patients with AML (14 females and 13 males) aged between 20 and 60 years (mean: 39.5 ±14.1 years) in this study; 52% of the subjects were female and 48% were male. All of the selected patients had white blood cell (WBC) counts of >10x10^9^/L and blasts of >4% in the peripheral blood. AML subtypes, according to the French- American-British classification, were 5 (19.2%) M1, 8 (30.8%) M2, 4 (15.4%) M3, 5 (19.2%) M4, and 4 (15.4%) M5. The blast counts in the specimens tested ranged from 5% to 71% with a mean value of 25% in peripheral blood. All AML cases were negative for terminal deoxynucleotidyl transferase (TdT). Seventeen out of 27 AML patients (63%) were found to be CD34-positive (mean CD34 expression: 60.13%), whereas 10 patients (37%) were CD34-negative. The control group ranged from 20 to 58 years of age (mean: 38 ± 10.6), of which 50% were male. All of the selected healthy controls had WBC counts of <10x10^9^/L.

**Gene Expression**

Comparison of VEGF-A and VEGF-C Expression between AML Patients and Normal Controls

**Figure 1:**
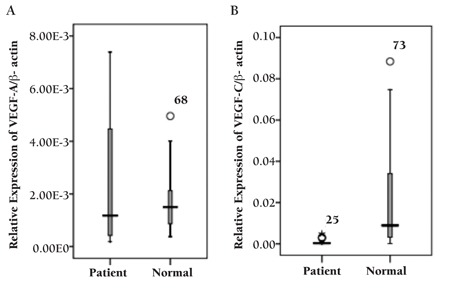
VEGF-A (A) and VEGF-C (B) mRNA expression in PBMCs of AML patients and normal controls (p=0.861 and p<0.001, respectively).

In spite of the wide range of individual values of VEGF-A or VEGF-C, median expression of VEGF-C mRNA in PBMCs of the control group were increased by about 43-fold compared to AML patients (p<0.001) (Figure 1). However, there were no significant differences in VEGF-A mRNA expression between leukemia cells and normal control cells (p=0.861) (Figure 1). There were also no significant correlations with PBMCs of VEGF-A and VEGF-C transcripts in patients and controls (rs=0.121, p=0.565 and rs=-0.170, p=0.438, respectively).

Association between VEGF-A or VEGF-C Expression in PBMCs of Leukemic Cells and Clinical Features

Assessment of correlation between gene expression levels of VEGF-A or VEGF-C and French-American-British subtypes, peripheral WBC count, percentage of blasts, absolute blast count, hemoglobin value, platelet count, age, and sex revealed no significant correlations.

Association between VEGF-A or VEGF-C Expression in PBMCs and Immunophenotype of AML Cells

We assayed the correlation between gene expression levels of VEGF-A or VEGF-C and expression of antigens CD2, CD3, CD4, CD7, CD10, CD11b, CD13, CD14, CD15, CD19, CD20, CD22, CD33, CD34, CD38, CD45, HLA-DR, TdT, and glycophorin A; no significant correlations were found.

Association between VEGF-A or VEGF-C Expression and Outcome

Further investigation was performed by using the Kaplan–Meier survival curve and log-rank test to evaluate the suitability of these molecules as prognostic factors. Patients were divided into a low group (expression of VEGF-A or VEGF-C below the median) and a high group (above the median). We rechecked the analysis based on the lower versus upper quartile (25%) of VEGF expression by developing a Cox model. All AML cases did not have bone marrow transplant. The median follow-up period of the 27 patients was 312 days (range: 1–990 days).

**Figure 2:**
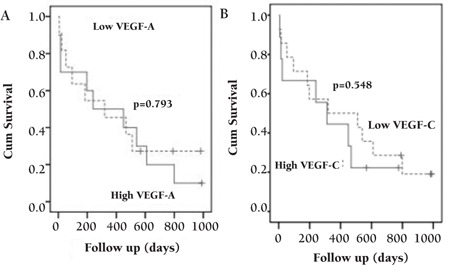
Kaplan–Meier survival curves for VEGF-A (A) and VEGF-C (B). Each angiogenesis factor was divided into high (solid line) and low (dotted line) concentration subgroups based on the distribution of mRNA levels.

We performed a univariate Cox regression analysis of the impact of VEGF-A and VEGF-C gene expression on overall survival. This analysis showed that neither sex and age nor WBC count, absolute blast count, hemoglobin level, platelet count, prothrombin time, and partial thromboplastin time were significantly related to prognosis in the study population. The Kaplan–Meier curves for overall survival stratified according to VEGF-A and VEGF-C expression in the PBMCs of AML patients are shown in Figure 2.

## DISCUSSION

The fact that angiogenesis may have an important role in AML and the key regulatory role of the VEGF/VEGFR complex in angiogenesis leads to the performing of studies regarding the role of VEGF in AML [26,27,28]. Fielder et al. reported that the leukemic cells of most patients with AML expressed VEGF-C [27]. Furthermore, Dias et al. demonstrated that VEGF-C, which was released from the endothelium, induced proliferation, promoted survival of AML cells, and protected VEGFR-3–expressing leukemic cells from chemotherapy-induced apoptosis [21].

In the present study, we observed a significant decrease of VEGF-C mRNA expression in leukemia cells compared to normal control cells (p<0.001). Although only 1 study has reported elevated expression of VEGF-C in the bone marrow of AML patients compared with the normal group [29], in 2 recent studies, in agreement with our results, decreased VEGF-C expression levels were reported in bone marrow mononuclear cells of AML patients compared to healthy controls. Lee et al. reported that the marrow level of VEGF-C was significantly lower (p<0.001) in AML patients compared to values in healthy controls [23]. Moreover, similar significant decrement of VEGF-C in the bone marrow of AML patients was observed by Hou et al. (p=0.0011) [24]. Loges et al. also reported that the expression of VEGF-C in AML patients was lower than in normal PBMCs; however, this difference was not significant [30].

In 2 previous studies, expressions of VEGF-C or VEGF-A were not an independent prognostic factor for relapse-free and overall survival [24,30], but it was shown that in the presence of higher levels of VEGF-C and VEGF-A, patients with high Ang-2 expression had a poor prognosis [24]. In this study, we found no relationship between VEGF-C expression levels and clinical outcome. It seems that angiogenesis is affected by different cytokines other than VEGF-C, as well as VEGF-C being affected by different cytokines

It has been shown that VEGF-A induces proliferation, survival, and protection of AML cells against apoptosis by an autocrine loop via VEGFR signaling [31,32,33].

In the present study, we did not observe any significant differences in VEGF-A mRNA expression of AML patients compared to controls (p=0.861), and this observation is in concurrence with a previous study that reported lower levels of marrow VEGF-A in AML compared to normal controls (p=0.158) [23]. However, these results are controversial with regard to previous studies that indicated the significantly enhanced expression of VEGF-A in AML patients’ bone marrow compared to controls [24,34]. Additionally, we did not observe any significant relationship between VEGF-A and clinical outcome.

Emerging evidence from genetically modified animal models,interestingly,proposes that elevated levels of VEGF-A may prevent, rather than promote, early tumor development and progression [35,36,37]. The study by Cervi et al. in a retrovirus-induced, spontaneous murine leukemia model reported a tumor inhibitory role for VEGF-A, and it was observed that a 2-fold overexpression of VEGF-A systemic levels leads to deceleration of tumorigenesis [37]. VEGF-A inhibits the growth and progression of various cancer types through recruitment of tumor inhibitory monocytic cells [38,39] and the negative regulation of tumor angiogenesis [35,36].

Stockmann et al. demonstrated that in the absence of myeloid cell-derived VEGF-A, an atypical high density vessel network is formed. They suggested that myeloid-derived VEGF-A plays a unique key role in facilitating changes in tumor vessel function and normalization [36]. Greenberg et al. observed that VEGF-A disrupts the function of vascular smooth muscle cells [35]. These studies have provided evidence to suggest that VEGF-A can act as a negative regulator of angiogenesis and tumor progression.

Overall, the studies described above suggest that VEGF-A plays a pivotal role in providing the mechanisms that regulate tumor growth and endow a survival advantage to the host. In addition, these studies propose that VEGF-A acts as an inhibitor of tumor growth when its levels are modulated through genetic modification before cancer induction. The dichotomous (enhancer or inhibitor) roles of VEGF-A in tumor angiogenesis are dependent on its concentration in the host microenvironment.

These contradictory results may suggest that a complex regulation of the cytokine system exists during the angiogenesis process in AML, and more studies are necessary to clarify the role of VEGF and other proangiogenic cytokines in this disease.

In conclusion, in our study, we observed a significant decrement of VEGF-C levels in the PBMCs of AML patients compared to healthy controls. However, there was no significant decrement in expression of VEGF-A mRNA of AML patients compared to the control group. We were not able to assess any role of VEGF-C or VEGF-A in predicting prognosis in AML patients by evaluating the VEGF expression of PBMCs. It seems that angiogenesis affects different cytokines other than VEGF-C or VEGF-A, and VEGF is also affected by different cytokines. Taken together, these findings help to provide new insights into the investigation of other angiogenic factors and cytokines that may play roles in the pathogenesis of AML. To clarify the role of VEGF in AML pathogenesis, further comprehensive studies with larger sample sizes are recommended.

**Acknowledgments**

We would like to acknowledge all AML patients and healthy individuals who vulnerably participated in this study.

**Conflict of Interest Statement**

The authors of this paper have no conflicts of interest, including specific financial interests, relationships, and/ or affiliations relevant to the subject matter or materials included.

## Figures and Tables

**Table 1:**
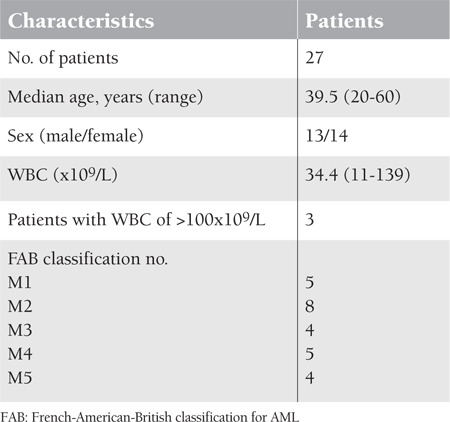
Characteristics of AML patients.

**Table 2:**
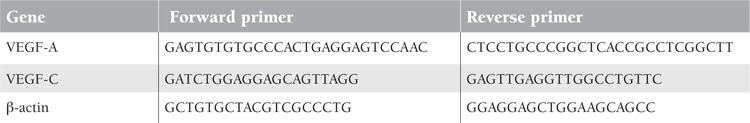
Sequences of primers.
